# 3D Contact Position Estimation of Image-Based Areal Soft Tactile Sensor with Printed Array Markers and Image Sensors

**DOI:** 10.3390/s20133796

**Published:** 2020-07-07

**Authors:** Jong-il Lee, Suwoong Lee, Hyun-Min Oh, Bo Ram Cho, Kap-Ho Seo, Min Young Kim

**Affiliations:** 1 HRI (Human Robot Interaction) Research Center, Korea Institute of Robotics and Technology Convergence, Pohang-si, Gyeongsangbuk-do 37553, Korea; banily07@kiro.re.kr (J.-i.L.); neoworld@kiro.re.kr (K.-H.S.); 2 School of Future Automotive & IT Convergence, Kyungpook National University, Daegu 41566, Korea; 3 Safety System R&D Group, Korea Institute of Industrial Technology, Daegu 42994, Korea; lee@kitech.re.kr (S.L.); jobbo@kitech.re.kr (B.R.C.); 4 School of Electronics Engineering, Kyungpook National University, Daegu 41566, Korea; ohm29@knu.ac.kr; 5 Department of Mechanical Engineering, Pohang University of Science and Technology, Pohang-si, Gyeongsangbuk-do 37673, Korea; 6 Research Center for Neurosurgical Robotic System, Kyungpook National University, Daegu 41566, Korea

**Keywords:** tactile sensor, image processing, soft material, printed array markers, camera, contact position

## Abstract

Tactile sensors have been widely used and researched in various fields of medical and industrial applications. Gradually, they will be used as new input devices and contact sensors for interactive robots. If a tactile sensor is to be applied to various forms of human–machine interactions, it needs to be soft to ensure comfort and safety, and it should be easily customizable and inexpensive. The purpose of this study is to estimate 3D contact position of a novel image-based areal soft tactile sensor (IASTS) using printed array markers and multiple cameras. First, we introduce the hardware structure of the prototype IASTS, which consists of a soft material with printed array markers and multiple cameras with LEDs. Second, an estimation algorithm for the contact position is proposed based on the image processing of the array markers and their Gaussian fittings. A series of basic experiments was conducted and their results were analyzed to verify the effectiveness of the proposed IASTS hardware and its estimation software. To ensure the stability of the estimated contact positions a Kalman filter was developed. Finally, it was shown that the contact positions on the IASTS were estimated with a reasonable error value for soft haptic applications.

## 1. Introduction

Tactile sensors have many uses and are expected to be beneficial in various fields. They will gradually be used not only for medical or industrial purposes but also as input devices and contact sensors for robots; tactile sensors for dexterous manipulation with robotics arms were developed by [1,2,3]. Tactile sensors used for measuring biological tissues, such as tumors, were reported by [4,5]. A soft tactile sensor was applied for tactile feedback to grasp the prosthetic limb [6]. Certain tactile sensors function as input devices for intuitive human–computer interaction [7]. If a tactile sensor is applied to various forms of human–machine interactions, not only is it important to have softness from the viewpoint of comfort and safety but it is also necessary for it to be easily customizable and inexpensive. Recently, there were several studies of tactile sensors that used soft material. Certain tactile sensors consisted of semiconductor transducers and soft materials, such as silicone rubber and polydimethylsiloxane [8,9,10,11]. They directly converted the applied force to an electrical signal based on electrophysical properties, such as the piezoelectric effect. However, as the softness of the soft material affects the sensitivity of the sensor signal and their performance, the material needs to be carefully selected and manufactured. These sensors also require a specific production process of their own, and their mass production cost is quite high even if their prototypes can be made at a relatively low cost. However, there are force/tactile sensors that use elastic materials whose deformation can be detected by the optical sensor [12,13,14]. These sensors have several light sources and photo-detectors that are covered by soft materials such as a silicone rubber. They convert the external force in a mechanical deformation into an electrical signal by using the photo-detectors. However, the special resolution of the sensor depends on the number of light sources and the photo-detectors. A tactile sensor that surrounds a conductive liquid-filled skin was also proposed [15,16]. This tactile sensor was capable of force, vibration, and temperature sensing, similar to the capabilities of the human touch. These sensory capabilities have been incorporated into the device without placing a single sensor in the skin. However, they require a specific conductive liquid; the complexly fabricated materials and their stiffness are difficult to change if customization is required according to their application. Recent research has produced a tactile sensor capable of measuring the force with a vector structure [17,18]. The sensor is composed of a complete embedded system and can be manufactured in a thin and compact form. The contact area where the external pressure was applied consisted of several layers of silicon. One of the multiple layers was a section filled with countless particles and was used as an element to express the force exerted from the outside. The sensor can measure the directional force, but it is not possible to measure the pressed depth or the contact position. In addition, despite the small allowable area, four cameras needed to be used, which was quite a disadvantage.

We developed an image-based soft tactile sensor using a small camera and general soft materials with attached array markers, which showed the deformations on the sensor surface [17,18,19]. Certain researchers have investigated image-based tactile sensors with soft materials [20,21,22,23,24,25,26]. The methods proposed in [20,21,22] require specific transparent elastic materials in which micro plastic beads for markers are exquisitely placed. The tactile sensor introduced in [23,24] needs to be filled with a specific fluid or water, which is carefully sealed with a silicon rubber and transparent plate. The tactile sensor proposed in [25,26,27] requires a fine array of pins molded on the inside of a silicon rubber skin. Most of the contact area of the existing tactile sensors use organic compounds such as silicone or synthetic rubber. In addition, to acquire tactile information, it has a common point that a precise electrical circuit design is required, or an expensive specific sensor must be used. Due to this feature, most tactile sensors must be tuned to be optimized for the environment. In addition, scalability may be degraded when upgrading to improve sensor performance and applying to various platforms.

The image-based tactile sensor that we proposed has the following three advantages. First, the various elastic materials, such as silicon rubber, textile, and artificial leather, can be used in the sensor if the markers can be attached or printed. Second, the shape, size, and texture are easily customized according to the application’s requirements by replacing the material. Third, it will be inexpensive because the specific production processes and equipment are not required. Apart from the advantages of hardware, it can be applied to various fields by utilizing the tactile sensor proposed by us. First, it can be applied as an input device capable of 3D input/output. In the case of general input devices such as a keyboard, mouse, tablet, and joystick, two-dimensional input methods are basically supported. The proposed sensor is an input/output device function within the platform in a 3D environment, and it can provide an immediate signal and receive feedback depending on the direction, force, or depth that the user presses. Through this, the robot or device that needs to be controlled can be controlled without dissimilarity, and in the event of an emergency, the user can be notified with a haptic function such as applying a force in the reverse direction or sounding a vibration. Second, it is possible to acquire the human body shape data. In general, a lot of equipment such as a motion capture system is used to extract human motion data. It can acquire high accuracy and various data, but if a person is lying down or leaning against a wall, the marker is obscured, and data measurement is impossible. In addition, it is difficult to acquire 3D human body shape data with a motion capture system. There is also pressure-sensor-based products that can be used in these situations. However, most likely only pressure data is acquired. In addition, there is a problem that the elastic force is poor and the resolution is low. If a person is lying or sitting on a platform that greatly expands the proposed tactile sensor, the human body curvature can be expressed as it is due to the fabric material characteristics of the elastic contact. In addition, information on force, depth, and direction can be acquired based on the high resolution of the image sensor. Furthermore, it has the advantage that it can be applied to various fields.

In this study, a new type of surface tactile sensor (IASTS) is proposed using a printed array marker and image sensor. Additionally, a method of estimating the contact position and depth using the proposed sensor will be described. Section 2 of this paper begins with an introduction to the prototypes of the proposed two models of IASTS and describes algorithms for image processing and feature extraction to estimate contact position and depth, the polynomial fitting algorithms, and the gaussian curve fitting algorithms. Section 3 conducts experiments to demonstrate what was introduced and described in Section 2. In Section 4, we will analyze the experiments performed in Section 3 and conclude in Section 5.

## 2. Materials and Methods

**Figure 1:**
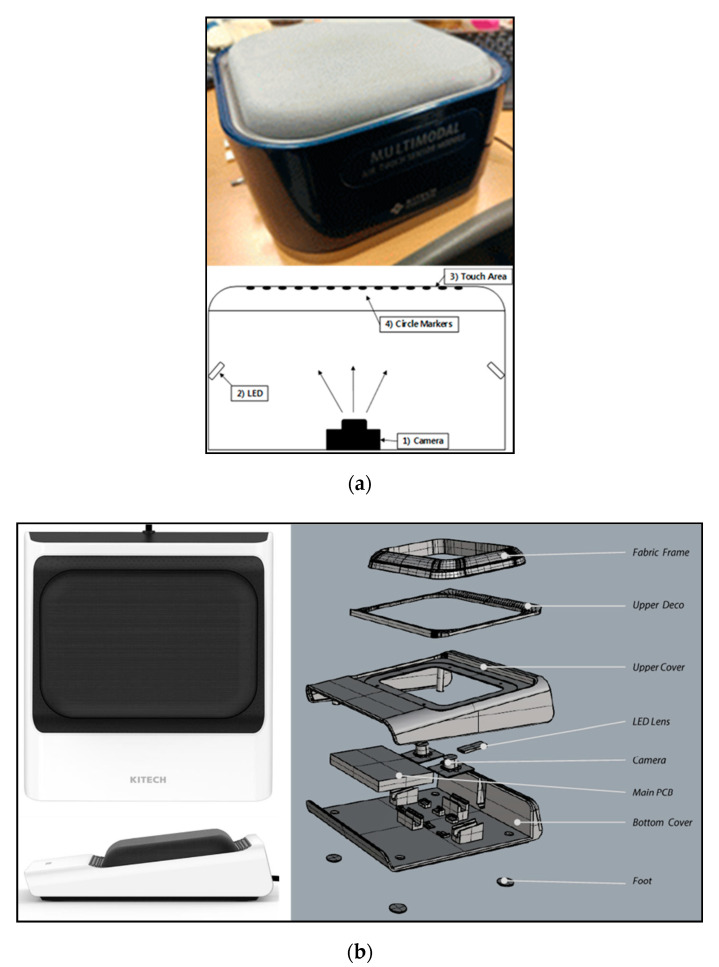
Two types of image-based areal soft tactile sensor (IASTS) (**a**) single-camera model and (**b**) dual-camera model.

The IASTS proposed in this paper is classified into two types of devices. It is classified into a model using the single-camera model and the dual-camera model. Figure 1 shows two models of IASTS proposed in this paper.

Figure 1a is a single-camera model of the IASTS prototype. This is the initial prototype model of the sensor proposed in this paper and is mainly used in environments with a small contact range. The contact recognition activation area is about 45 × 60 mm. In the top of Figure 1a, the prototype IASTS using the single-camera model is shown. In the bottom of Figure 1a, the internal structure of the sensor is indicated. When the user presses the contact area (3), the circular marker (4) attached to the lower portion of the upper plate moves according to the pressed position or direction. The number of markers attached to the single camera model is 117 (13 × 9) in total. The image sensor (1) located at the bottom acquires data by recording the circular marker (4) attached to the lower portion of the top plate. The material used for the contact area (3) is a material through which light does not completely pass, and the inside of the sensor is completely dark. To recording the circular marker (4) with the image sensor (1), light is required, but the LED (2) attached to the side plays a role.

Figure 1b is a dual-camera model of the IASTS prototype. The prototype has a size of 153 ×165 × 48 mm and is ergonomically designed; it has an oblique shape that supports the wrist. The contactable area of the sensor was 133 × 91 mm. The material of the contact portion was soft; therefore, there was a possibility that the frame holding the contact portion would be bent. To prevent this, we used a high-strength metal frame. To make the IASTS thin, the electronic components were optimally placed. In the left side of Figure 1b, the prototype using the dual-camera model is shown. The right side of Figure 1b shows the modeling. The operation method is like the single-camera model. However, by increasing the number of image sensors from 1 to 2, it has the advantage of high resolution and wide recognition range. In addition, the number of markers increased by about 1.5 times as compared to the existing, the contact recognition area was expanded, and the thickness was reduced by about 1/2. The total number of markers attached to the dual camera model is 154 (14 × 11). It is also characterized by being designed and manufactured ergonomically so as not to strain the wrist. The advantage of the proposed IASTS is that it has a simple structure as shown in Figure 1, but it is free in size expansion and can be applied to various parts. The description of the algorithm for acquiring related data when the user uses IASTS is described in the next section.

### 2.1. Contact Position and Depth Estimation Algorithm

**Figure 2:**
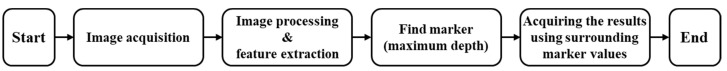
The sequence of data processing to determine contact position and depth.

IASTS cannot acquire a signal from the contact surface like an established pressure sensor or tactile sensor. To obtain the contact position and depth values, the motion of a circular marker attached to the inside is captured by an image sensor, and then a series of data processing is performed to obtain results. The sequence of data processing is shown in Figure 2.

First, an image including the entire marker is acquired using an image sensor. Second, the features of each marker are extracted through image processing. Third, tracking the marker with the maximum depth value. To obtain the depth value of each marker, a polynomial fitting algorithm is applied. Finally, precise contact position and depth values are obtained by applying the tracking marker and surrounding marker data. The algorithm used here is a Gaussian fitting algorithm. The detailed description of the method of extracting the features of each marker through the image processing, the polynomial fitting algorithm, and the Gaussian curve fitting algorithm will be described again below.

### 2.2. Image Processing and Feature Extraction

**Figure 3:**
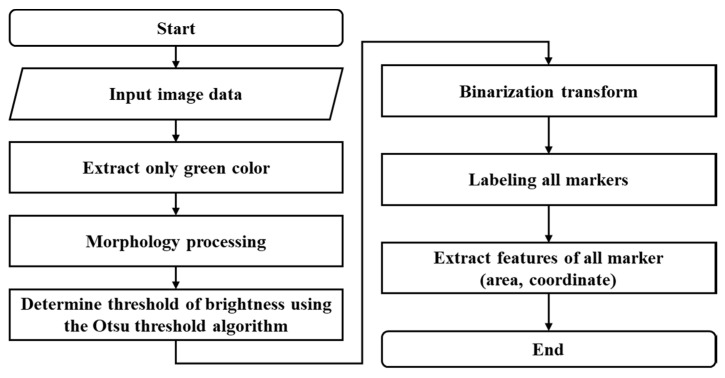
Input image processing diagram.

Figure 3 shows the image processing to extract features from the acquired image. First, the original input image from the image sensor was subjected to image preprocessing procedures to extract the features of markers, as shown in Figure 1.

To shorten the processing time, the three red–green–blue channel images were converted into one channel image. The markers attached to this hardware system were green; therefore, a single image was generated by choosing the green channel only for a good contrast image between the background and the marker. Then, the image noise was canceled by morphological operations, and binarization was performed to separate the background and the marker. To do this, we used the Otsu algorithm to determine a threshold for making a black image or a white image from the original image. The Otsu algorithm finds an appropriate threshold value for the brightness of the input image. When the image pixels are categorized into two classes based on the distribution of the histogram, there are two classes that minimize the dispersion within the class or maximize the dispersion between the classes. The internal structure of the IASTS is closed, and the lighting to illuminate the markers takes on a critical role to image the marker shape and intensity. Therefore, the accuracy of the marker detection is increased by acquiring an optimal threshold value by using the Otsu algorithm. The binarized image is subjected to a labeling process for all the markers. When the image preprocessing process is completed, the area and coordinate values of all the markers can be obtained.

Unlike the single-camera model, the dual-camera model must combine two separate screens. We used a primitive method to combine the two screens. In order to prevent the markers from falling within the range of each screen when the markers located on the outside are pressed, the method of combining the two cameras by grasping and pasting the intermediate positions of the overlapping sections was applied.

**Figure 4:**
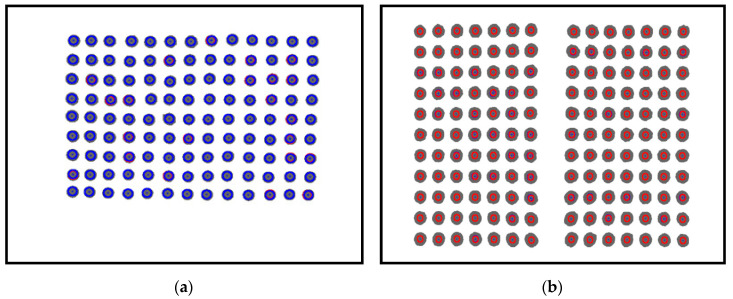
The result of image processing (**a**) single-camera model and (**b**) dual-camera model.

Figure 4 shows the results obtained based on the image processing.

Figure 4a shows the result of a single-camera model. The blue circle means the marker recognized using labeling, the blue dot means the position of the marker, and the gray circle inside the blue circle means the real area of the filtered marker. Figure 4b shows the result of the dual-camera model, as in the single-camera, the red circle is the marker recognized using labeling, the blue dot is the location of the marker, and the gray circle inside the blue circle is the real area of the filtered marker. This means when a contact occurs on the contact surface based on the features obtained from the image processing process, data such as a movement direction, a movement distance, and a contact depth of each marker can be acquired.

### 2.3. The Polynomial Fitting Algorithm

To find the marker with the maximum depth value, we first need to know the depth information of each marker. To acquire the depth value based on the data of each marker’s feature (area, coordinate) acquired in Section 2.2, preprocessing is necessary. In the case of the single-camera model IASTS, the distance of the marker located in the front cannot be obtained because a single camera sensor is used. Therefore, it is necessary to calibrate the depth value using the feature data of the marker, and this paper solves it by applying the polynomial fitting algorithm.

**Figure 5:**
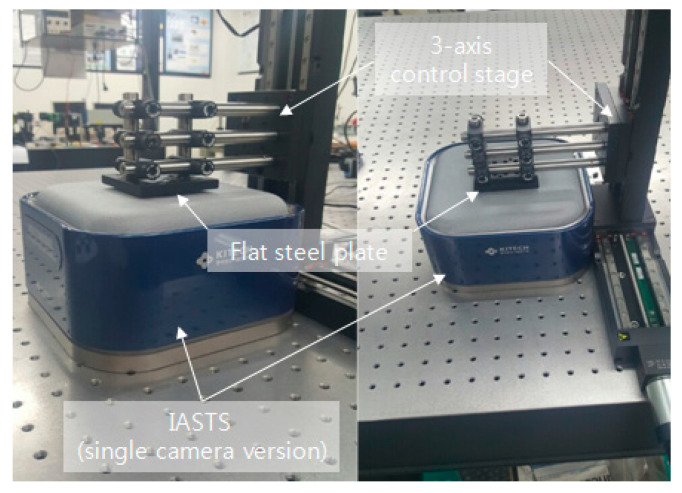
The experimental environment based on three-axis control stage for feature data extraction to perform the polynomial fitting algorithm.

Figure 5 shows the experimental environment for acquiring data to perform the polynomial fitting algorithm. In the figure, a structure surrounding the entire contact surface labeled flat steel plate is attached to the 3-axis control stage. Then, by controlling the stage, feature data of a marker that changes for each depth (in 1 mm increments) are extracted. Next, the polynomial fitting algorithm is used to generate a polynomial set of feature data and depth values for each marker. Here we have concluded through several experiments that the third-degree polynomial is the best fit. When the feature data of the markers input in real time is input using the finally generated polynomial set, the depth value of each marker can be derived.

The dual-camera model IASTS is equipped with two camera sensors and can perform the stereo calibration to obtain the distance of the marker located in front. However, in the case of the dual-camera model IASTS proposed in this paper, the stereo calibration process is not performed because the purpose is to simply increase the contactable area. Additionally, since the polynomial fitting algorithm is applied by performing the same experiment as the single camera model IASTS, the content of the dual camera model is omitted in the relevant section.

**Figure 6:**
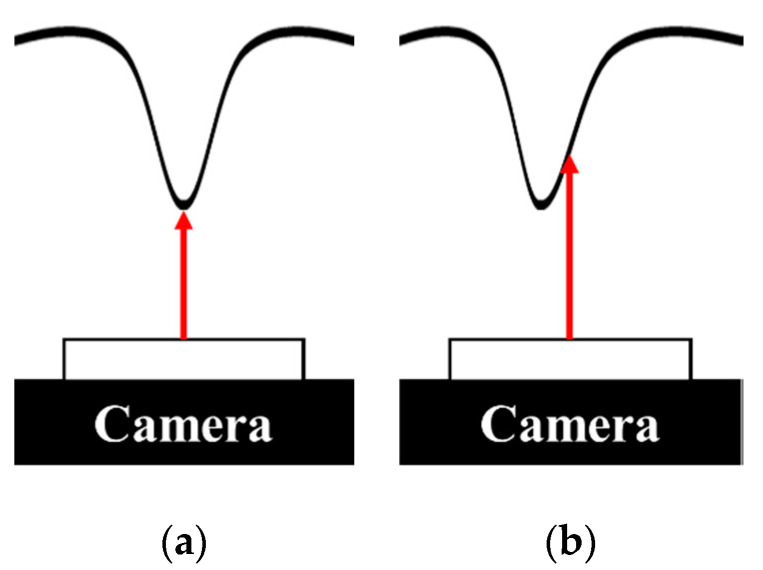
Correlation of contact surface change with camera sensor according to contact position (**a**) when pressing the center of the contact surface (**b**) when pressing the outer of the contact surface.

**Figure 7:**
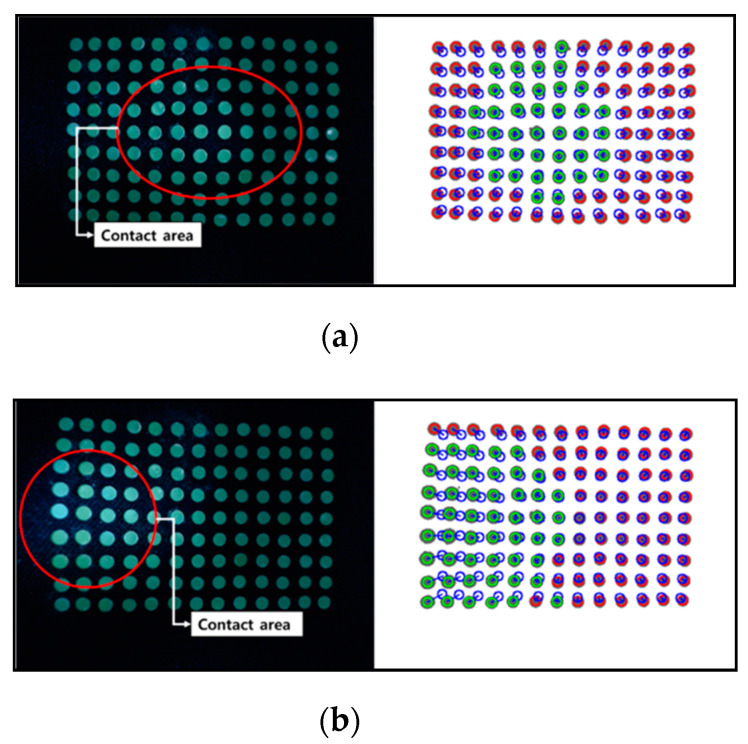
Identifies the tendency to change the features of the marker according to the contact location (**a**) the camera view when pressing the center of the contact surface (**b**) the camera view when pressing the outer of the contact surface.

Figure 6 and Figure 7 show the relationship between marker area and coordinate displacement when the contact surface of IASTS is pressed. In the figure, the blue circle means the initial position of the marker, the green circle is the marker recognized as a contact, the red circle is the marker recognized as a noncontact, and the blue line connected between the blue circle and the green circle indicates the coordinate displacement from the initial position of the marker.

The image sensor is attached to the middle of the sensor. When pressing the center of the contact surface as shown in Figure 6a, the camera shows the result as in Figure 7a. Markers located in the vertical vicinity of the camera lens rarely change position and only change the area. However, as shown in Figure 6b, if the outer surface of the contact surface is pressed, the camera shows the result as in Figure 7b. It can be seen that as the distance from the vertical direction of the camera lens changes, the position and the area change simultaneously. Therefore, when applying the polynomial fitting algorithm, polynomials for two cases must be generated.

**Figure 8:**
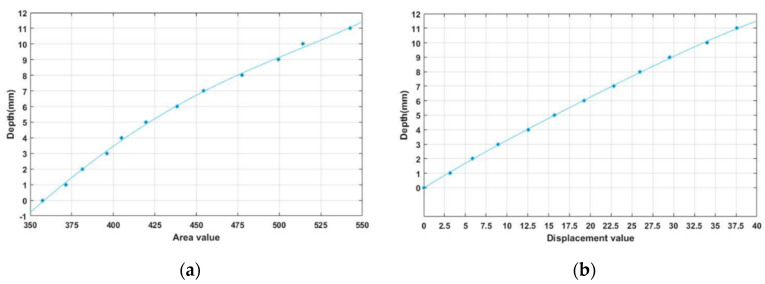
Area data along depth changes and its polynomial fitting (**a**) the result of third-order polynomial fitting to the area (**b**) the result of third-order polynomial fitting to the coordinate displacement.

Figure 8 shows the results of applying the polynomial fitting algorithm for depth based on data obtained using a 3-axis control stage. Figure 8a shows the result of third-degree polynomial fitting to the area, and Figure 8b shows the result of third-degree polynomial fitting to the coordinate displacement.

Equations (1) and (2) give the polynomial for each case to obtain the depth value. $a_{a},\;b_{a},\;c_{a},\;\mathrm{and}\;d_{a}$ are polynomial fitting coefficients for the area of the marker for each order, and $a_{c},\;b_{c},\;c_{c},\;\mathrm{and}\;d_{c}$ are polynomial fitting coefficients for the coordinate displacement of the marker for each order. $x_{a}$ is the area value of the marker, $x_{c}$ is the coordinate displacement value of the marker, $y_{a}$ is the depth value obtained using the marker area value, and $y_{c}$ is the depth data obtained using the coordinate displacement value of the marker.

(1) $$a_{a}x_{a}^{3}+b_{a}x_{a}^{2}+c_{a}x_{a}+d_{a}=y_{a},$$

(2) $$a_{c}x_{c}^{3}+b_{c}x_{c}^{2}+c_{c}x_{c}+d_{c}=y_{c},$$

**Figure 9:**
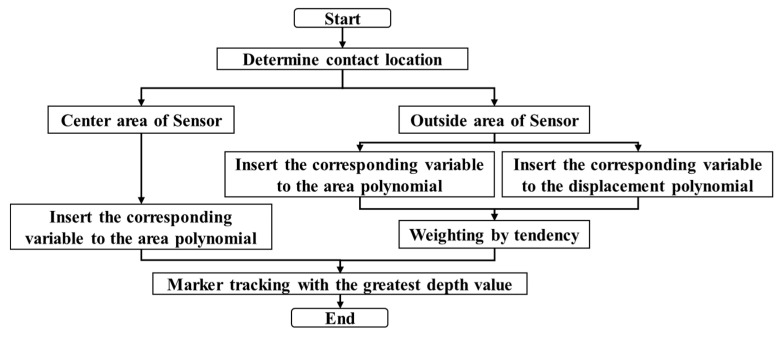
Marker position estimation diagram from polynomial fitting process.

Figure 9 shows a flow chart for tracking markers with maximum depth values. As mentioned in Figure 6 and Figure 7, the amount of change in the area of the marker or the difference in coordinate displacement depends on the contact position. Weights are used to distinguish them. The closer the actual contact position is to the vertical position of the camera, the more weight is given to the amount of change in the area of the marker, and the greater the distance, the more weight is given to the coordinate displacement value of the marker.

**Figure 10:**
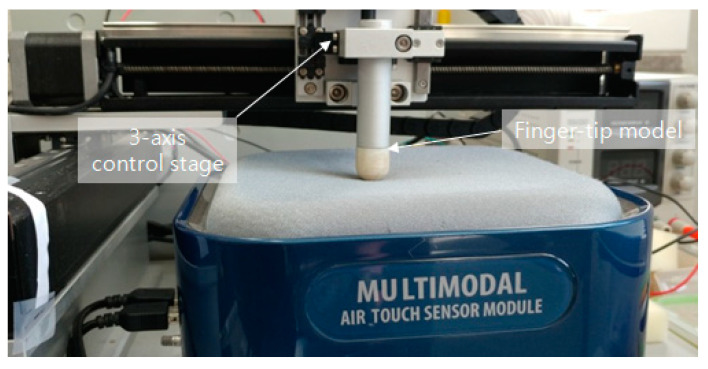
Experimental environment for obtaining weighted data sets.

Figure 10 shows the experimental environment configured to obtain weights for the features of each marker. To obtain a depth value that can be estimated using Equations (1) and (2), a control stage that can be moved in a 3-axis direction is used. A finger-tip was attached to the contact portion. Depth values for area and displacement were obtained by pressing each depth from the marker located at the top left to the marker located at the bottom right. The experiment was conducted dozens of times.

Equations (3) and (4) refer to the process for obtaining the standard deviation of the area and displacement of the marker. The reason for using the standard deviation is that reliability can be determined for each condition when experiments are performed in the same environment multiple times based on different conditions.

(3) $$\sqrt{\left(\frac{\sum_{i=0}^{n}{\left(y_{ia}-y_{r}\right)}^{2}}{n}\right)}=\sigma_{a},$$

(4) $$\sqrt{\left(\frac{\sum_{i=0}^{n}{\left(y_{ic}-y_{r}\right)}^{2}}{n}\right)}=\sigma_{c},$$

$y_{ia}$ is the value for $y_{a}$ in Equation (1) in the i-th experiment, $y_{ic}$ is the value for $y_{c}$ in Equation (2) in the i-th experiment, and $y_{r}$ means the actual depth value. $\sigma_{a}$ is the standard deviation for the area, and $\sigma_{c}$ is the standard deviation for the displacement. Through the above process, each marker has a standard deviation data set for area and displacement.

To obtain the weight of each marker, the standard deviation-based weight obtained in Equations (3) and (4) is calculated using Equation (5).

(5) $$\frac{\sigma_{c}}{\sigma_{a}+\sigma_{c}}=w_{a},\frac{\sigma_{a}}{\sigma_{a}+\sigma_{c}}=w_{c},$$

(6) $$w_{a}y_{a}+w_{c}y_{c}=y,$$

(7) $$w_{a}+w_{c}=1,$$

$w_{a}$ is a weight for an area change amount of the corresponding marker, and $w_{c}$ is a weight for a coordinate displacement of the corresponding marker. Using this, the final depth value in Equation (6) can be obtained using the feature data of each marker input in real time. $y_{a}$ and $y_{c}$ represent the results derived from Equations (1) and (2), and y represents the final depth value of the corresponding marker. Equation (7) is a condition for satisfying Equation (6).

**Figure 11:**
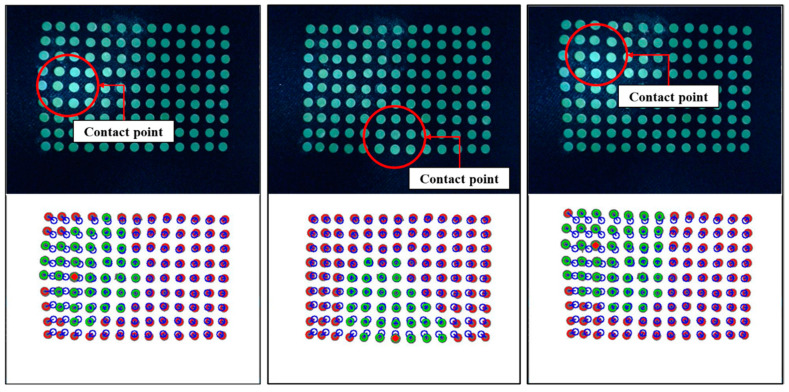
Maximum depth value marker tracking results using polynomial fitting algorithm.

When the depth value of each marker is obtained from Equation (6), the marker having the maximum depth value, which is the last step in Figure 9, is traced, so that the marker closest to the contact position can be known. The output as shown in Figure 11 is displayed when an arbitrary position is pressed. The red circle at the bottom of Figure 11 represents the marker with the maximum depth value.

### 2.4. The Gaussian Curve Fitting Algorithm

In Section 2.3, the polynomial fitting algorithm was used to obtain the marker with the maximum depth value, but it is difficult to confirm the precise contact location with only the information. Therefore, to improve accuracy, a Gaussian curve fitting algorithm is performed using the marker data around the corresponding marker.

**Figure 12:**
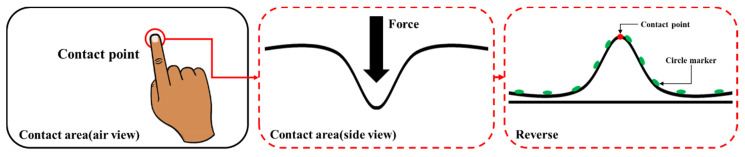
Basis for application of Gaussian fitting algorithm.

The reason for applying the Gaussian curve fitting algorithm to this paper is shown in Figure 12. When pressed at any position on the contact surface as shown in the left side of Figure 12, if viewed from the side as shown in the middle of Figure 12, the contact surface increases in a recessed form. It has the shape of a bell. As shown in the right side of Figure 12, when the shape is flipped, it is like the shape of the Gaussian graph. Assuming that a plurality of circular markers attached to the contact surface are respective points constituting a Gaussian graph, Gaussian curve fitting can be performed using coordinate values of each marker and a peak point serving as a contact point can be obtained. If the Gaussian curve fitting algorithm is not applied, when the marker is not located at the actual contact position, the marker having the largest depth value can be found, but an error is likely to occur.

**Figure 13:**
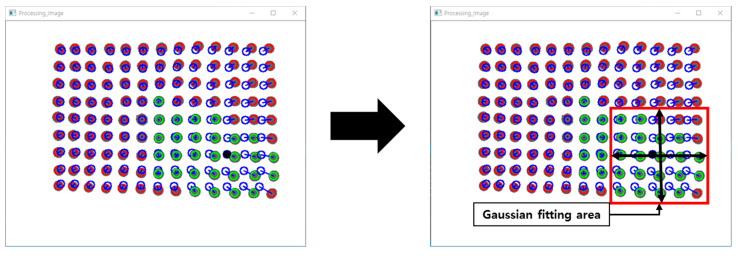
Target areas for Gaussian fitting algorithm.

Define the maximum depth marker as a reference marker and perform Gaussian curve fitting for the horizontal and vertical axes, respectively, as shown in Figure 13. The information of the markers that are separated up to the second line in the up, down, left, and right directions of the reference marker is used.

(8) $$f\left(x\right)=aexp\left(-b{\left(x-c\right)}^{2}\right),$$

(9) $$ln\left(y\right)=ln\left(a\right)-b{\left(x-c\right)}^{2}=\left[ln\left(a\right)-bc^{2}\right]+2bcx-bx^{2},$$

(10) $$ln\left(y\right)=d_{1}+d_{2}x+d_{3}x^{2},$$

(11) $$b=-d_{3},\;c=-\frac{d_{2}}{2d_{3}},\;a=exp\left[d_{1}-\frac{d_{2}^{2}}{4d_{3}}\right],$$

Equation (8) means a general Gaussian function. x represents the coordinates of each peak point (depth), and f(x) represents the height value for the x coordinates. When x=c, f(x) can obtain the maximum depth value a. Therefore, the purpose is to find a and c. To use the variables in the exponential function, convert them to logarithmic form on both sides to replace them with Equation (9). When curve fitting ln(y) of Equation (9) to a quadratic function of x, it can be expressed as Equation (10), and variables $d_{1},\;d_{2},$ and $d_{3}$ can be expressed by Equation (11).

(12) $$ln\left(y\right)=\left[\begin{array}{c} d1\\ d2\\ d3 \end{array}\right]\left[\begin{array}{c} 1 \end{array}\;x\;x^{2}\right],$$

(13) $${\left[\begin{array}{ccc} 1 & x_{i-2,j} & x_{i-2,j}^{2}\\ 1 & x_{i-1,j} & x_{i-1,j}^{2}\\ 1 & x_{i,j} & x_{i,j}^{2}\\ 1 & x_{i+1,j} & x_{i+1,j}^{2}\\ 1 & x_{i+2,j} & x_{i+2,j}^{2} \end{array}\right]}^{-1}\left[\begin{array}{c} \begin{array}{c} ln\left(y_{i-2,j}\right)\\ ln\left(y_{i-1,j}\right) \end{array}\\ ln(y_{i,j})\\ ln(y_{i+1,j})\\ ln(y_{i+2,j}) \end{array}\right]=\left[\begin{array}{c} d1\\ d2\\ d3 \end{array}\right],$$

To apply Equation (10) in the current three-dimensional environment, change it into a matrix form as Equation (12) and replace it with Equation (13). Here, $x_{i,j}$ is a coordinate of the reference marker, and $y_{i,j}$ is a depth value of the reference marker. i is the horizontal direction, j is the vertical direction, and Equation (13) uses the four-marker information located on the left and right based on the horizontal direction to find the variable for the horizontal direction. In the case of obtaining the vertical coordinates, the value of i in Equation (13) is fixed and the variable is obtained by changing the value of j. If the variables $d_{1},\;d_{2},$ and $d_{3}$ are obtained through Equation (13), c and a can be calculated by substituting in Equation (11), and the maximum depth value for the contact position can be estimated.

## 3. Results

### 3.1. Experiment of IASTS (Single-Camera Model)

**Figure 14:**
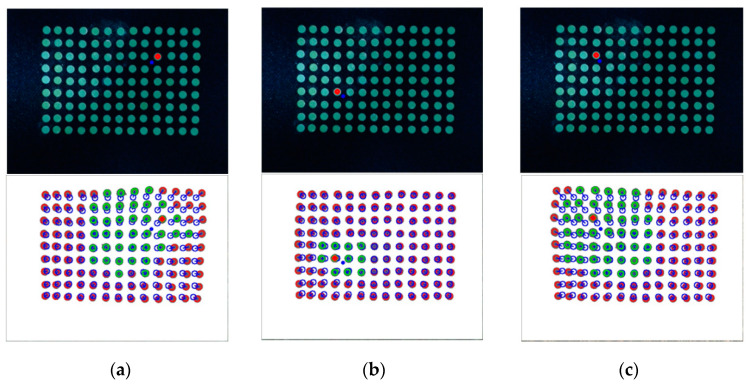
Contact position estimation by using a gaussian fitting process (**a**) the results of contact position estimation when pressing the upper right (**b**) the results of contact position estimation when pressing the bottom left (**c**) the results of contact position estimation when pressing the upper left.

Experiments and verifications are performed using the IASTS of the single camera model proposed in this paper and the algorithm of the three routines proposed in Section 2. The experiment is performed using the three-axis control stage equipped with the finger-tip used in Figure 10. Figure 14 shows the results of estimating a contact position with a blue dot by performing Gaussian fitting on the neighborhood markers located around the marker having the largest depth value.

Table 1 also shows the data of the actual contact position, estimated contact position through the Gaussian fitting, actual contact depth value, and the estimated depth value in the cases of (**a**), (**b**), and (**c**).

The actual contact position and contact depth were determined quantitatively by using a three-axes control stage. Table 1 shows the numerical data of the contact position estimated through the Gaussian fitting of Figure 14 with its actual values. In addition, three experiments were conducted at the same position. The average error distance and depth through the algorithm was within 3.4 mm in the XY position and 1.8 mm in the Z position.

### 3.2. Experiment of IASTS (Dual-Camera Model)

**Figure 15:**
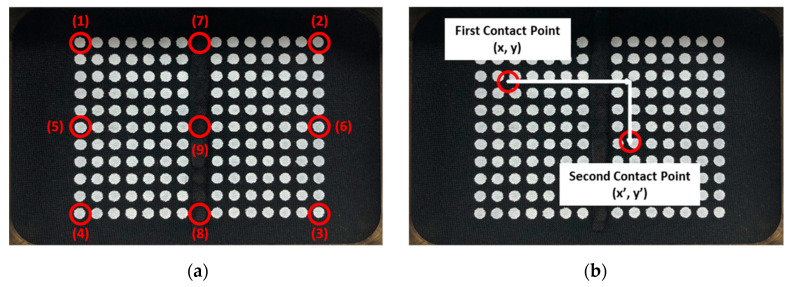
Experiment method of IASTS. (**a**) 1st experiment rule (**b**) 2nd experiment rule.

As in the single camera model, experiment and verification are conducted using the three-axis control stage equipped with a finger-tip shown in Figure 10. However, the experimental method is different from the single camera model. To do this, two types of experiments were conducted. Figure 15a is the first experiment. After specifying an arbitrary position, the contact surface of IASTS was pressed using a three-axis linear stage, and the results were compared and analyzed. The numbers (1) to (9) seen in Figure 15a specify the contact sequence and contact location. Figure 15b is the second experiment. After specifying an arbitrary position, the contact surface of IASTS was pressed and released using a three-axis linear stage. Then, the results were compared and analyzed when the contact surface was pressed and released again after moving a predetermined distance. According to the experimental plan in Figure 15a, a contact experiment was performed using a three-axis linear stage, and the coordinate data for estimating the contact position was obtained. In addition, the contact depth estimation data was obtained by pressing the depths of 1, 2, and 3 mm at each position in Figure 15a.

**Figure 16:**
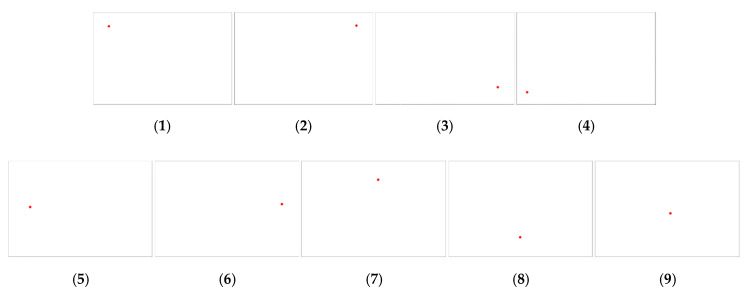
Experiment 1 for proposed system and algorithm verification (**1**) when pressing the upper left corner (**2**) when pressing the upper right corner (**3**) when pressing the bottom right corner (**4**) when pressing the bottom left corner (**5**) when pressing the left (**6**) when pressing the right (**7**) when pressing the upper (**8**) when pressing the bottom (**9**) when pressing the center.

The results of estimating the contact position for each contact position are shown in (1) to (9) in Figure 16. Each image in Figure 16 represents the basic interface representing the contact location of IASTS. For the convenience of division, only the calculated contact position was marked with a red dot, and no other information was displayed.

The resulting data for Figure 16 can be seen in Table 2 and Table 3. Table 2 analyzes the actual contact position coordinates and the estimated contact position coordinates. The average error distance for the estimation results of the contact position coordinates of the entire experiment was approximately 3.1 for the X axis and approximately 4.4 for the Y axis. The error rate was approximately 3.56% for the whole case. Table 3 analyzes the actual contact depth and the contact depth estimation result. The error depth for the overall estimated depth result was approximately 0.06 mm. The error rate was approximately 3.83% for the whole case.

**Figure 17:**
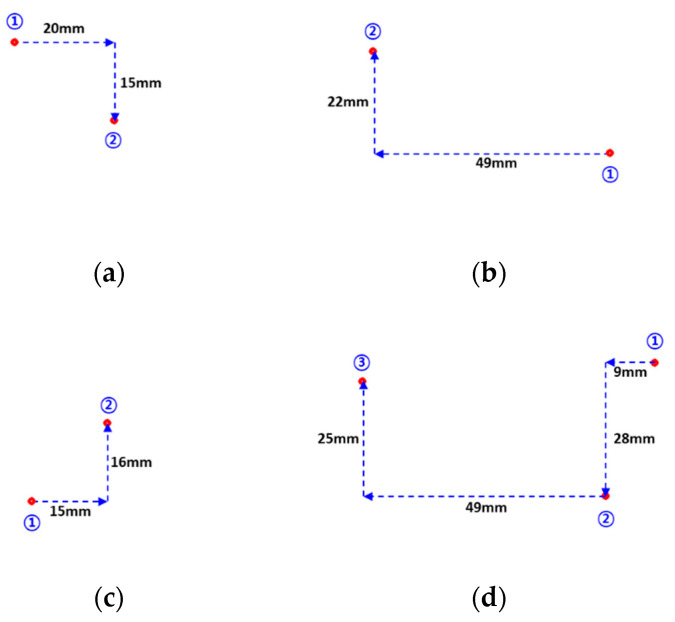
Experiment 2 for proposed system and algorithm verification (**a**) 1st experiment path (**b**) 2nd experiment path (**c**) 3rd experiment path (**d**) 4th experiment path.

Figure 17 shows the results according to the experimental plan in Figure 15b. It compares and analyzes the results when a contact is made after a quantitative value is moved based on a specific location. In Figure 17a–d, the numbers 1, 2, and 3 indicate the order in which the IASTS contact surfaces were pressed. Additionally, the number near the dotted arrow indicates the distance traveled in millimeters. Table 4 shows the experimental results in Figure 17. The estimated contact coordinates of the experiments (a), (b), (c), and (d) were classified into two categories X and Y. The actual moving distance of the linear stage was expressed to obtain the moving value of the pixel according to the actual moving distance. The average of the moving values of the coordinates according to the distance in 1 mm units was approximately 10.28.

## 4. Discussion

We conducted an experiment to verify the proposed contact position and depth estimation algorithm using two types of sensors (single- and dual-camera models).

First, the data obtained through the experiment in Figure 13 was analyzed using the single-camera model IASTS. The average error distance for the estimation results of the contact position coordinates of the entire experiment was approximately 1.675 mm, and the standard deviation was approximately 1.08 mm. The average error depth for the contact depth estimation result of the whole experiment was approximately 0.84 mm, and the standard deviation of the error was approximately 0.64 mm. The IASTS of the single-camera model was verified so that the error range was relatively small.

Second, the data obtained through the experiments in Figure 16 and Figure 17 were analyzed using the dual-camera model IASTS. In relation to the experiment in Figure 16, the average error distance for the estimation results of the contact position coordinates of the entire experiment was approximately 6.3, and the standard deviation was approximately 2.5. The average error depth for the contact depth estimation result of the whole experiment was approximately 0.06 mm, and the standard deviation of the error was approximately 0.04 mm. In the experiment shown in Figure 17, by obtaining the coordinate values per meter per 1 mm, the average standard deviation was approximately 10.28, and the standard deviation was 0.53. Assuming that the coordinate value per meter per 1 mm was 10, the average error distance of the experiment in Figure 16 was 0.63 mm, and the standard deviation was approximately 0.25 mm.

There were three reasons for the relatively small error in the contact position of the dual-camera IASTS as compared with the single-camera IASTS. The first reason was the hardware difference. The dual-camera IASTS model was based on a single-camera IASTS because it included various factors, such as the contact surface material, camera mount position, lighting arrangement, and light intensity adjustment. The second reason was the number of markers covering the contact surface. The dual-camera IASTS had a larger number of markers than the single-camera IASTS; therefore, the effective range of contacts was relatively wide. The third reason was the difference in resolution. The dual-camera IASTS featured two image sensors. When the resolution increased, the amount of change of the marker could be accurately measured and predicted.

## 5. Conclusions

This paper describes the structure and principle of a single-camera-type IASTS and dual-camera–type IASTS and introduces the contact position and depth estimation algorithms. To verify the reliability of the proposed system, we conducted and proved data acquisition experiments and analysis. Various haptic systems can be built based on the proposed IASTS. Further, the expressions related to tactile sense in an augmented reality/virtual reality environment are also possible. Additionally, the algorithms can be applied to haptic devices of various sizes by customizing the hardware. In our future studies, we will develop a deep learning-based tactile position measurement algorithm to improve precision. Functions, such as motion recognition and text recognition through specific gestures, will be introduced.

## Figures and Tables

**Table 1:** Actual contact information and estimated contact information (Unit: mm).

Experiment	Real Contact		Estimated Contact		Error Distance		Real Depth Value	Estimated Depth Value	Error Depth
	X	Y	X	Y	X	Y			
(a)	43	13	43.14	14.29	0.14	1.29	10	10.76	0.76
			42.66	14.12	0.34	1.12		10.79	0.79
			42.35	14.44	0.65	1.44		10.65	0.65
(b)	14	28	16.54	30.11	2.54	2.11	7	7.2	0.2
			16.75	29.95	2.75	1.95		7.15	0.15
			16.41	29.84	2.41	1.84		7.15	0.15
(c)	16	11	15.41	14.31	0.59	3.31	13	14.52	1.52
			15.36	14.22	0.64	3.22		14.81	1.81
			15.52	14.33	0.48	3.33		14.52	1.52

**Table 2:** Contact coordinate position comparison and error analysis (unit: pixel).

Experiment	Real Contact		Estimated Contact		Error Distance		Error Rate (%)	
	X	Y	X	Y	X	Y	X	Y
1	107	70	104	76	3	6	2.80	8.57
2	841	70	846	77	5	7	0.59	10.00
3	841	580	846	590	5	10	0.59	1.72
4	107	580	115	590	8	10	7.48	1.72
5	107	325	116	322	9	3	8.41	0.92
6	842	325	845	321	3	4	0.36	1.23
7	475	70	480	79	5	9	1.05	12.86
8	475	580	467	590	8	10	1.68	1.72
9	475	325	479	320	4	5	0.84	1.54

**Table 3:** Contact depth comparison and error analysis (unit: mm).

Experiment	Real Depth	Estimated Depth	Error Depth	Error Rate (%)
1	1.00	0.89	0.11	11.00
	2.00	1.91	0.09	4.50
	3.00	2.89	0.11	3.67
2	1.00	0.85	0.15	15.00
	2.00	1.89	0.11	5.50
	3.00	2.92	0.08	2.67
3	1.00	1.09	0.09	9.00
	2.00	2.11	0.11	5.50
	3.00	3.13	0.13	4.33
4	1.00	1.07	0.07	7.00
	2.00	2.08	0.08	4.00
	3.00	3.08	0.08	2.67
5	1.00	1.03	0.03	3.00
	2.00	2.01	0.01	0.50
	3.00	2.98	0.02	0.67
6	1.00	1.06	0.06	6.00
	2.00	1.98	0.02	1.00
	3.00	2.97	0.03	1.00
7	1.00	1.05	0.05	5.00
	2.00	2.04	0.04	2.00
	3.00	3.01	0.01	0.33
8	1.00	1.03	0.03	3.00
	2.00	2.02	0.02	1.00
	3.00	3.04	0.04	1.33
9	1.00	0.98	0.02	2.00
	2.00	1.97	0.03	1.50
	3.00	2.99	0.01	0.33

**Table 4:** Analysis of contact location along the travel path.

Experiment		1st Contact Position (Pixel)	2nd Contact Position (Pixel)	Moving Distance (mm)	Coordinate per 1 mm
(a)	X	259	466	+20 mm	10.35
	Y	215	377	+15 mm	10.80
(b)	X	712	220	−49 mm	10.04
	Y	420	210	−22 mm	9.55
(c)	X	305	466	+15 mm	10.73
	Y	503	341	−16 mm	10.13
(d_1)	X	845	744	−9 mm	11.22
	Y	181	460	+28 mm	10.37
(d_2)	X	744	236	−49 mm	9.96
	Y	460	219	−25 mm	9.64
